# The Pattern and Impact of Hospital-Acquired Infections and Its Outlook in India

**DOI:** 10.7759/cureus.48583

**Published:** 2023-11-09

**Authors:** Jay Thakkar, Amardeep Shanoo, Sajal Gupta, Aditi Thakkar

**Affiliations:** 1 Department of Ophthalmology, Jawaharlal Nehru Medical College, Datta Meghe Institute of Higher Education and Research (Deemed to be University), Wardha, IND; 2 Department of Obstetrics and Gynaecology, Jawaharlal Nehru Medical College, Datta Meghe Institute of Higher Education and Research (Deemed to be University), Wardha, IND; 3 Master of Public Health, National Institute of Mental Health and Neurosciences, Bengaluru, IND

**Keywords:** nosocomial infections, covid-19, hospital-acquired infections, antibiotics, medical icu

## Abstract

Nosocomial infections form one of the most challenging tasks to deal with in a hospital setting. The burden of hospital-acquired infections (HAI) significantly affects the patient's cost of medical treatment and seriously impacts the economy of a developing country like India. Haphazard antibiotic use for the treatment of these infections has led to the development of resistance among the microbes, and factors that complicate microbial eradication further worsen the scenario. A large percentage of the HAI are preventable by simply following up various protocols which when supported by judicious antibiotic use can declutter the severity of the problem. Organized infection control measures, trained hospital staff, and continuous surveillance of HAI in healthcare settings will help deal with nosocomial infections. Although the ability to deal with HAI in a patient might determine his survival after acquiring a nosocomial infection, prevention remains the best option at all times. Lowering down the burden of nosocomial infections is of utmost importance since it contributes significantly to the overall resource utilization of the hospital and the country. Implementing the use of nanoparticles and nanotechnology in delivering target-specific drugs might be helpful in preventing antibiotic resistance. Taking into account reports of nosocomial infection patterns in various centres of India, the seriousness and consequences of HAI are uncovered in this article.

## Introduction and background

Nosocomial infections or hospital-acquired infections (HAI) are the one which present within 48 hours of hospital admission, within three days of discharge, or postoperatively within a month [1]. The infection should not be active or incubating during admission; in other words, the infections should be acquired during contact with healthcare [1,2]. About 10-20% of admitted patients acquire nosocomial infections in India [3]. Intensive care units (ICU) remain the primary site for HAI, contributing to a quarter of all hospital infections [4]. In a clinical setup, *Staphylococcus aureus* is the most common cause [5]. Poor hygiene practices and conditions, plus the increased hospital patient load, significantly contribute to the same [6]. Treating bacterial infections overwhelmingly with non-susceptible antibiotics without prior testing is a chief opportunity for the selective proliferation of resistant bacteria due to acquired mutations [5]. The severity of the disease and repeated exposure to treatment procedures among ICU patients form the basis of repeated infections in these patient groups [7]. Studies show that nosocomial transmission of neonatal infections, a major cause of neonatal mortality in India, promotes the development of more antimicrobial-resistant strains [8]. The infection source can be endogenous or exogenous, but an exogenous source is more common. Older populations and those immunocompromised, including patients with diabetes and patients with drains and intubation, are more susceptible [4,6].

Ventilator-associated pneumonia (VAP), intravenous line-associated bloodstream infections (BSI), and catheter-related urinary tract infection (UTI) remain among the customary HAI [9]. Long duration of surgery, nutrient deficiency, cardiac cachexia, and hypothermia add to the same, which, in addition, adds to the management cost estimate with still increased mortality rate [10]. Surgical site infections (SSI) are the third most commonly reported HAI in India, as per the National Nosocomial Infections Surveillance System (NNIS) [11]. At times, intrinsically nonpathogenic organisms, if immunocompromised, present as HAI. Nosocomial infections can occur as an outbreak or as an endemic [12]. Intelligibly, infections acquired by healthcare personnel are nosocomial [13]. Though ICU count up less than 10% of the total bed occupation in most hospitals, 20% of nosocomial infections occur in ICU [4]. The rate and prevalence of nosocomial infections and grades of resistance vary greatly with topography and in different ICU [14]. In terms of revenue, one with low- and mid-income countries has added the burden of HAI [15-17]. Studies show that HAI causing prolonged ICU stays were associated with increased treatment costs by two times [18]. It is also seen that 15 of every 100 admitted patients acquire one nosocomial infection and one in every 10 of these succumbs to death [19]. This is attributable to the fact that multidrug-resistant infections need high-end antibiotics, prolonged hospital stays, and more diagnostic tests [20]. A high percentage of 23.6% is attributed to crude mortality due to HAI [21]. Furthermore, it isn't possible to eradicate the HAI; prevention, early detection, and control of spread set out to be the best measures.

## Review

Methodology

A literature search was conducted to comprehensively review the data regarding nosocomial infections and its outlook in India. Multiple electronic databases, including PubMed, MEDLINE, Embase, and Google Scholar, were searched using the following keywords and combinations: nosocomial infections, hospital-acquired infections, COVID-19, medical ICU, and antibiotics. The search encompassed articles published from 1993 to 2023. In addition to electronic database searches, reference lists of relevant articles and review papers were manually screened to identify additional studies. The inclusion criteria involved selecting observational studies, experimental studies, systematic reviews, and meta-analyses that examined the association between nosocomial infections and its outlook in India. Studies with adult human participants were included. There was no exclusion criteria. Only peer-reviewed, published articles were considered for inclusion. Independently, reviewers assessed the titles, abstracts, and full-text articles for eligibility, with any discrepancies resolved through discussion and consensus. The comprehensive literature search aimed to ensure the inclusion of relevant studies and provide a thorough analysis of the nosocomial infections in India.

Dealing with nosocomial infections

One-third of the HAI can be prevented by preventive measures itself; surveillance and prevention form the foundation to the management of nosocomial infection. Surveillance involves the collection, interpretation, and analysis of health-related data as collected and reported by various agencies like the Antimicrobial Resistance Surveillance and Research Network of the Indian Council of Medical Research (ICMR) and National Centre for Disease Control (NCDC). Despite preventive measures, HAI continue to occur. De-escalation, at its simplest, refers to treatment with an opening dose of a broad-spectrum antibiotic until before the susceptibility results are at hand. This is to be restrained for critical patients; doing it without reason would compound the resistance pattern among the bacteria. Over time, treatment of an infection with the same antibiotic in a setup builds up the selection pressure favouring the evolution of resistance. Rotation refers to the temporary dropout of an antibiotic or an entire class in a system to turn down or stabilize the resistance figure; this, in the case of ICU, isn't significant to much extent. Uncalled de-escalation therapies in patients recently have called an increased accumulation of resistant bacteria [5]. If therapy to manage them isn't evolved at pace, the scenario may continue to worsen. Prevention, however, always proves to be better than cure, hence the need to limit HAI. Considering the pattern of HAI prevalence at various tertiary care hospitals in India and management practices, the seriousness and consequences of HAI are to be considered.

How we look to manage the spread of HAI depends on how rigorously we know about the causative organism and its close track of how we devise strategies to manage the same. The UK national surveillance service handles one pronounced project, compounding the data of reported HAI into one database, helping us to keep track of the way we are moving in HAI control and the primarily involved organisms during a given period. The survey in 2002 concluded that intravenous catheters were the most common source of bacteremia [5]. As much as 40% of HAI can be controlled by the sanitation of bad hygiene equipment like protection from body fluids, use of gloves, masks, and aprons mainly by healthcare workers, tunnelling the catheters, and preferring the subclavian vein over the internal jugular and femoral vein. Relevant antibiotic therapy has a noteworthy effect, while isolation of the affected identified patients adds to the control [5]. Judicious but targeted antibiotics, once the susceptibility pattern is known, help reduce selection pressure; routine surveillance of ICU by experienced microbiologists may add to a decrease in the same load.

Who is affected with nosocomial infection?

Patient Vulnerability

Basically, this refers to intrinsic traits that inheritably compel the patient to develop the disease. This directs us to take a preventive turn if in case the patient is immunocompromised or at a higher risk. The Acute Physiology and Chronic Health Evaluation (APACHE) and diagnosis-related groups are the available indices that can help us assess how critical is the pre-presented illness.

Interventional Equipment and ​Extraneous Factors

**Figure 1:**
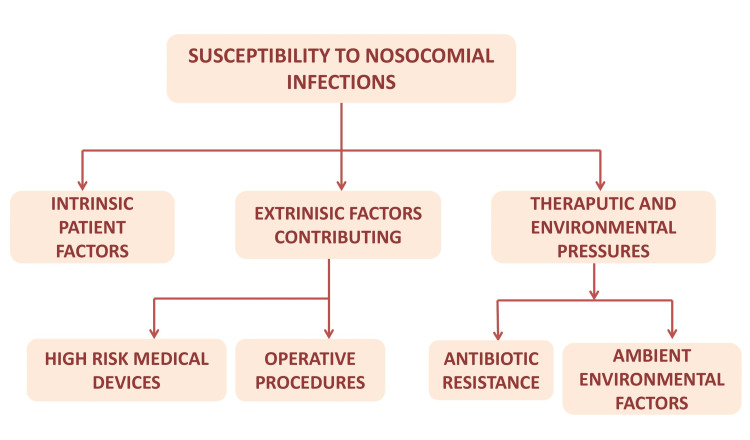
Susceptibility to nosocomial infections

The treating hospital and the handling personnel themselves may be an add-on to the risk of HAI. Using medical devices for treatment acts as a vehicle for the entry of these organisms in the body from the surroundings or from within the body to another part. Hence, the intervention duration with these is set according to need and centrally devised and institutionally devised policies. SSI are also a crucial burden to HAI management. Others, like environmental factors, also have a mild role to play [12] (Figure 1).

The prevalence

A group of pathogens, namely, *Enterococcus faecium*, *Staphylococcus aureus*, *Klebsiella pneumoniae*, *Acinetobacter baumannii*, *Pseudomonas aeruginosa*, and *Enterobacter* species (ESKAPE), cause the majority of HAI [22]. Overall, *Escherichia coli* and *Staphylococcus aureus* remain the most prevalent among HAI, but their chief prevalence sites are contrasting [12]. Recent reports of resistance are increasingly seen among gram negatives like *Klebsiella*, *Pseudomonas*, and *Enterobacter*. Because of the wide spectrum of available drugs, the concern over resistance development has spurred up. In the case of *Klebsiella*, the plasmid-mediated sulfhydryl reagent variable (SHV1) gene shows dramatic variations, therefore adding to the resistance. Among the *Enterobacter* species, gradual uplift of chromosomal type 1 beta-lactamase has led to the development of resistance among the wide-spectrum covering drugs including imipenem. ICU isolates are more of the target than non-ICU. The high cost of research studies and new drug development by pharmacological companies, though a continuous process, is the chief driver of the market costs in the healthcare system. The newer agents would be primarily more expensive than the older ones, therefore restricting the prescription of the same to all the infected ones. Developing a new drug as the older one gets resisted won't be the solution as ensuring a sufficient range of spectrum of antibiotics is a must [12].

Nosocomial infections, coronavirus disease 2019 (COVID-19), and the overall control

COVID-19 is an acute severe respiratory infection caused by severe acute respiratory syndrome coronavirus-2 (SARS-CoV-2). The first half of COVID-19 was associated with an overburdened hospital and healthcare system [23]. COVID-19 showed a balanced effect on HAI, the positive sides being the use of personal protective equipment, hand hygiene, and distancing. The negative impact was seen due to the overuse of antibiotics [24,25]. Methicillin-resistant *Staphylococcus aureus* (MRSA) has remained a burden organism to deal with during the pandemic. MRSA is considered the leading cause of death as far as antimicrobial resistance is concerned [26]. The longer duration of hospital stay associated with severe COVID-19 infection was responsible for causing superadded bacterial coinfections particularly in those requiring prolonged mechanical ventilation and those admitted in ICU [24]. COVID-19 was associated with increased use of immunomodulators and steroids which reduces pulmonary inflammation but predisposes the patient to further infections [25]. The specified authorities in a hospital form the chief base in its control. The lab personnel in the hospital must also take up with the decided steps to achieve the desired goals. The mere formation of a committee and directing the rules don't help. Its members will be actively working according to the directions, which would need skill training and knowledge to perform activities for infection control. Now, the United States follow a strategy of appointing an infection control practitioner. Physician hospital epidemiologists serve as the directors of infection control programs [12]. The Joint Commission on Accreditation of Healthcare Organizations (JCAHO) provides the broad elements that drive infection control. It has defined certain indicators to check on the hospital's performance. The Centers for Disease Control and Prevention (CDC) has organized some early training for hospital epidemiologists and integrated care providers. To ensure the strength of the control program, it should not fail to ensure (1) organized control measures and activities, (2) trained control staff, and (3) reporting system about SSI to surgeons.

Types, agents, distribution, and surveillance

There are 14 types of HAI as per CDC, of which medical device-associated HAI are the most common which is of bacterial etiology in 90% of cases [27]. The contribution pattern by individual bacteria is variable and changing over time, for example, the incidence levels due to *Streptococcus* and coagulase-negative and coagulase-positive *Staphylococcus* retook the position in being more involved in HAI, and the incidences of *Klebsiella pneumoniae* and *E. coli* declined between the 1960s and 1980s [6]. A study from Japan reported that gram-negative anaerobes contribute the heaviest load in primary infections, with *Enterococcus* being the second most culprit, followed by *Streptococcus* and *Staphylococcus* among the gram-positive aerobes and *E. coli*, *Klebsiella*, *Pseudomonas*, and *Enterobacter* among the gram-negative aerobes [13]. 

S. aureus

Normally, 20% have normal colonization with *S. aureus*; immunocompromised patients are maximally susceptible. The infection may involve superficial and deep tissues; toxin-mediated diseases include food poisoning and toxic shock syndrome due to enterotoxin and toxic shock syndrome toxin 1 (TSST-1). Transmission is through the skin and fomites. 

E. coli

A gram-negative bacteria which is mainly involved in the causation of UTI, septicemia, pneumonia, and meningitis mediated by endotoxins, type 3 secretion systems, and the bacterial capsule. The infection is transferred from person to person through the environment, food, and water.

Vancomycin-Resistant Enterococci

They form the normal female genitourinary flora and are subject to cause infection in surgery patients using their hemolysin, surface protein gelatinase.

Klebsiella

It is the colonizer of the gastrointestinal tract (GIT) and causes septicemia and pneumonia mediated by their endotoxins, cell wall, and polysaccharide. Infection is through contaminated hospital equipment and in between infected persons [13,28,29]. 

Pseudomonas

It causes HAI with high mortality involving the renal, urinary, and respiratory tract. Incubators and sink soaps act as sites of fomite contamination [13].

Clostridium difficile

It serves as the typical colonizer and a cause of HAI and is responsible for causing colitis; its spores even do resist disinfection. The working staff has a pivotal role in its transmission [13]. There is a recent trend in the increase of *Clostridium difficile* contribution in the causation of HAI [30] (Table 1). Surveillance simply refers to collecting and interpreting the nosocomial data that would help us determine and interpret the healthcare practice. The collected trends and data allow us to direct the hospital authorities to take appropriate measures to ensure quality healthcare delivery. The microbiology lab has a pivotal role in adding a striking edge to the surveillance of HAI by improving the quality of results when supported well by strategies designed by the directives of infection control programs. High-performance labs, ensuring that the lab member is part of a surveillance program, trained staff, and strict lab result monitoring with judicious lab resource use would be of valuable significance [12].

**Table 1:** Organisms attributed in the causation of nosocomial infections

Nosocomial infection	Organism attributed
Urinary tract infection	E. coli
	Proteus
	Klebsiella
	Enterobacter
	Enterococcus
Respiratory tract infection (pneumonia)	Staphylococcus
	Pseudomonas
	Haemophilus influenzae
	Acinetobacter
Gastrointestinal infection	Clostridium
	Enterococcus
	Rotavirus
Surgical site infection	Staphylococcus
	Pseudomonas
	Enterococcus
Bloodstream infection (septicemia)	Staphylococcus
	E. coli
	Klebsiella

Epidemiological insights and trends

The study of HAI prevalence requires data collection from the desired patient group, that is, whose duration of ICU stay exceeds 48 hours. Further, it requires grouping of the patients according to the site of infection, type of infection, microorganism involved in its causation with its antibiotic susceptibility, patient's age group, differentiation of the infection as being nosocomial or not by microbiological as well as clinical findings, APACHE score, environmental sampling from floors, walls, and all the sites potentially contaminated with patient's fomites, recording of all medical interventional techniques used on the affected patient like catheters and intubations, and finally data analysis in comparison to the control group of patients who are not affected [31-33]. A retrospective analysis of medical ICU reports at Smt. Kashibai Navale Medical College and General Hospital in Pune, India, was conducted over a year's time frame; of the 537 records, 366 case reports with a stay duration of more than 48 hours were evaluated. 9.6% (32 patients; 35 infections) of these developed nosocomial infections, with respiratory tract infections (RTI) being most common, followed by UTI. *Acinetobacter* was the most common cause of RTI, 21% of which were multidrug-resistant. Resistance prevalence among *Pseudomonas* counted to 7.1%, the second most common causative of RTI and the most important cause of UTI. Maximally, penicillins were used as a treatment modality, with the mean ICU stay duration being 16.5 days for patients affected with HAI in contrast to a short of 6.4 days for those who were not, with respective mortality rates of 28.1% and 31.2% [31].

The results of another study conducted between July 2014 and December 2015 reported that 87 of the 157 enrolled patients developed nosocomial infection. Most affected patients had comorbidities, and *Klebsiella* and *E. coli* were the predominant cause. The patients' length of stay varied, that is, 15.24 ± 10.9 and 4.46 ± 5.03 days for the affected and non-affected ones, respectively, and a percentage of 39 patients developed more than one nosocomial infection [32]. In an 18-month-long study conducted at a tertiary care centre in Karnataka, India, 37 of the 355 ICU patients were found to have been affected with HAI, 55.2% of which were UTI followed by ventilator-associated and catheter-related infections that counted to 35.78% and 11.52%, respectively. *Klebsiella pneumoniae* and *Pseudomonas aeruginosa* were the most predominant causes of UTI and RTI, which also were chiefly found in hospital environment sampling [33]. A cardiac surgery ICU study from All India Institute of Medical Sciences (AIIMS) in Delhi, India, between January 2013 and December 2014 reported 7156 admissions, of which 6864 satisfied the inclusion criteria to be a part of the study. Three hundred nineteen patients developed HAI. Results of the study showed that one-third of the total admitted patients died. Duration of hospital stay, requirement of mechanical ventilation, and patients with cyanotic heart diseases were at higher risk of infection. RTI was the most commonly reported infection, followed by SSI, BSI, and UTI [4].

Coping strategies and advancements

The World Health Organization (WHO), in 2014, declared antimicrobial resistance as a threat to global health security [34]. Data availability about HAI needs to be improved [35]. Recently, a five-year action plan on antimicrobial resistance, an important component of the Ministry of Health and Family Welfare (MoHFW), Government of India, called as Antimicrobial Resistance Surveillance and Research Network, has been uplifted by ICMR and NCDC in 2013 and 2014, respectively. It ensures the account of 25 laboratories around the country that continuously report the susceptibility and resistance pattern of various highly resistant pathogens. The strict surveillance by trained experts, including by the apex medical centres of the country like Jai Prakash Narayan Apex Trauma Center of AIIMS that develop and apply the protocols along with the data from high-standard labs, ensures the generation of high-quality data in regard to HAI. Monitoring also has to ensure the practices in medical device interventions, with an overtime pivot aim of broadening institutional capacity for infection control by vigorous task force training and further widening the strength of existing programs; this ultimately will nourish the strength with which how effectively we respond to the HAI outbreaks. Currently, this all has a limited scale. The rapid expansion of the same with an aim to include all private and public health centres is what we aspire for. Certification and clearance from the National Accreditation Board for Hospitals and Healthcare Providers (NABH) and National Health Mission (NHM) add up to the surveillance power of HAI in the country. Overall, the buildup of patient safety is what remains the priority and the target of the designed programs [9].

Nanotechnology has emerged as a potential way to drug delivery and improve therapeutics. Recent advancements include the inclusion of nanoparticles, especially the metallic ones owing to their high stability, and to increase the bacterial membrane permeability resulting in increased antibiotic uptake by the bacterial cells, altering bacterial signal transduction, causing free radical damage, and blocking oxidative phosphorylation. Nanomedicine is an emerging branch in nanotechnology that aims to offer high target-specific treatment, deliver drugs with poor absorption and solubility, and facilitate prolonged drug delivery preventing biofilm formation [27]. Table 2 depicts the summary of all the studies included in the article. 

**Table 2:** Summary table of studies included in the review HAI: hospital-acquired infections; UTI: urinary tract infection; ICU: intensive care unit; LRTI: lower respiratory tract infections; SSI: surgical site infections; CDC: Centers for Disease Control and Prevention; MRSA: methicillin-resistant *Staphylococcus aureus*; ICMR: Indian Council of Medical Research; NABH: National Accreditation Board for Hospitals and Healthcare Providers; BSI: bloodstream infections; COVID-19: coronavirus disease 2019; CLABSI: central line-associated bloodstream infection; VAE: ventilator-associated event

Authors	Year	Findings
Chahardehi and Ghasemi [1]	2023	Though all HAI cannot be avoided, adopting hospital guidelines to deal with the same is necessary. Newer modalities like using antimicrobial coatings with chemicals like cyclodextrin may be tried and adapted.
Singh et al. [2]	2023	There is a difference in risk factors for the development of HAI between the patients who undergo and who do not undergo surgery.
Goyal and Chaudhry [3]	2019	Continuous primary education about the precautions in preventing nosocomial infections is expected to significantly reduce morbidity and mortality due to the same.
Mythri and Kashinath [4]	2014	Use of invasive devices significantly increases the risk of nosocomial infections especially among the geriatric age group. UTI and pneumonia remain the most common infections in ICU.
Inweregbu et al. [5]	2005	Gram-positive organisms are one of the leading causes of nosocomial infections, more than gram negatives. Inadequate therapy is associated with increased antimicrobial resistance. Knowing the resistance profile of the organism is important prior to the initiation of treatment.
Saba and Balwan [6]	2023	Surgical procedures pave a way in the causation of nosocomial infections.
Girou et al. [7]	1998	Study result shows that in an ICU setup, a higher therapeutic activity after day 3 of depressed consciousness is associated with a higher risk of nosocomial infections.
Robinson et al. [8]	2023	Prior maternal assessment for organism colonization doesn’t show any relation with neonatal BSI.
Edwardson and Cairns [9]	2019	One-third of the HAI are preventable. Intensivists encounter twice the number of these patients due to higher use of medical devices.
Sahu et al. [10]	2016	Gram-negative bacillary LRTI is the most common nosocomial infection after a cardiac surgery.
Kumar and Rai [11]	2017	A multidisciplinary approach taking care of pre-, intra-, and postoperative factors is needed to prevent SSI.
Emori and Gaynes [12]	1993	Infection control programs should chiefly focus on the prevention of nosocomial infections. Involving the microbiology department in active infection surveillance at hospital level and data collection about the causative organisms and their antimicrobial profile might help in the control.
Khan et al. [13]	2015	Need to devise infection control programs at the hospital level in addition to CDC recommendations in an attempt to achieve the desired infection control.
Akhtar [14]	2010	*P. aeruginosa*, *K. pneumoniae*, and *E. coli* followed by MRSA and *S. aureus* are most commonly attributed in the causation of nosocomial infections in medical ICU.
Swaminathan et al. [15]	2017	Role of various health bodies like ICMR and NABH and their action plans in the surveillance of nosocomial infections.
Murhekar and Kumar [16]	2022	The overall risk of nosocomial infection is higher by up to 20 times in low- to middle-income countries.
Mathur et al. [17]	2022	The result of one-month surveillance shows higher incidence of BSI with *Klebsiella* being the most common cause and UTI with *Candida* as the most common attributable organism among the participated hospitals.
Chacko et al. [18]	2017	Nosocomial infection acquired during ICU stay almost doubled the cost of treatment for the patient and also prolonged the duration of hospital stay.
Mateescu et al. [19]	2023	The pathogenic organisms present in the community influence the hospital microbial environment.
Raoofi et al. [20]	2023	The prevalence of nosocomial infection is increasing globally being highest in Central Africa, the attributed organism is mainly *E. coli* in the South East Asia region, the highest prevalence is in transplant wards followed by neonatal ICU, and the maximum incidence is in lower-income countries.
Chandra et al. [21]	2023	Mortality associated with nosocomial infections is twice as high as with community-acquired infections; hence, there is a need to know the antimicrobial profile of the hospital.
Chakraborty et al. [22]	2023	Use of a combination of a bactericidal and a bacteriostatic drug during empirical therapy would help to reduce antimicrobial resistance.
Rosenthal et al. [23]	2022	The COVID-19 pandemic was associated with increased CLABSI and VAE.
Biondo et al. [24]	2023	COVID-19 infection predisposes patients to viral infection due to impaired immune response as a result of viral infection.
Ramasamy et al. [25]	2022	Rate of nosocomial infection is similar in patients treated with immunomodulators or with standard care protocol in COVID-19 patients.
Samia et al. [26]	2022	Proposition of using MRSA as a marker to measure the efficacy of an infection control program in a health setting.
Ananda et al. [27]	2022	Need to establish the role of nanoparticles for the treatment of nosocomial infections owing to developing microbial resistance.
Neog et al. [28]	2021	Although outbreaks of *Klebsiella oxytoca* are lower, they are associated with increased mortality among those immunocompromised.
Sharma et al. [29]	2023	Increasing antimicrobial resistance trend among the *Klebsiella pneumoniae* to higher antibiotics.
Sinha and Behera [30]	2023	Increasing trend of *Clostridium difficile* in the causation of nosocomial infections.
Pradhan et al. [31]	2014	Nosocomial infections were associated with an increase in hospital stay duration but not the mortality.
Choudhuri et al. [32]	2017	Age more than 60 years, male gender, associated comorbidities, mechanical ventilation, and longer duration of stay are associated with higher rates of nosocomial infections.
Shalini et al. [33]	2010	There is a high rise in nosocomial infections caused by gram negatives like MRSA.
Das et al. [34]	2023	There is an increasing resistance trend to third-generation cephalosporin and carbapenems among the chief organisms causing nosocomial infections.
Bheemavarapu et al. [35]	2018	There is a lack in quality and quantity of data about the nosocomial infection trend in India.

## Conclusions

HAI significantly contribute to patients' mortality chances in an ICU setup and the unnecessary healthcare burden. Hence, it is essential to consider all the aspects and factors preventing its occurrence in any healthcare setup. Designing and follow-up of infection control programs at the national, state, and hospital levels and their coordinated functioning are the need of the hour. Including a microbiology laboratory in all aspects of management and surveillance would be very helpful in epidemiologic studies of the infection burden at the hospital level. Prescribing antibiotics after sensitivity testing, at all possible times, would positively impact HAI control and avoid drug resistance. Overall, prevention should remain the baseline strategy at all times and in all settings in dealing with HAI.
